# BMP2 promotes proliferation and invasion of nasopharyngeal carcinoma cells via mTORC1 pathway

**DOI:** 10.18632/aging.101230

**Published:** 2017-04-28

**Authors:** Meng-He Wang, Xiao-Min Zhou, Mei-Yin Zhang, Lu Shi, Ruo-Wen Xiao, Li-Si Zeng, Xian-Zi Yang, X.F. Steven Zheng, Hui-Yun Wang, Shi-Juan Mai

**Affiliations:** ^1^ State Key Laboratory of Oncology in South China, Guangzhou, 510060, China; ^2^ Collaborative Innovation Center for Cancer Medicine, Sun Yat-Sen University Cancer Center, Guangzhou, 510060, China; ^3^ Zhoukou Hospital of Traditional Chinese Medicine, Zhoukou, China; ^4^ Cancer Center of Guangzhou Medical University, Guangzhou, China; ^5^ Rutgers Cancer Institute of New Jersey, Rutgers University, New Brunswick, NJ 08901, USA

**Keywords:** nasopharyngeal carcinoma, BMP2, metastasis, mTORC1

## Abstract

Bone morphogenetic protein-2 (BMP2) is a secreted protein that highly expressed in a variety of cancers and contributes to cell proliferation, migration, invasiveness, mobility, metastasis and EMT. However, its clinical significance and biological function in nasopharyngeal carcinoma (NPC) remain unknown up to now. Up-regulation of BMP2 was first observed in NPC cell lines by a genome-wide transcriptome analysis in our previous study. In this study, BMP2 mRNA was detected by qRT-PCR and data showed that it was upregulated in NPC compared with non-cancerous nasopharynx samples. Immunohistochemistry (IHC) analysis in NPC specimens revealed that high BMP2 expression was significantly associated with clinical stage, distant metastasis and shorter survival of NPC patients. Moreover, overexpression of BMP2 in NPC cells promoted cell proliferation, migration, invasiveness and epithelial-mesenchymal transition (EMT). Mechanistically, BMP2 overexpression increase phosphorylated protein level of mTOR, S6K and 4EBP1. Correspondingly, mTORC1 inhibitor rapamycin blocked the effect of BMP2 on NPC cell proliferation and invasion. In conclusion, our results suggest that BMP2 overexpression in NPC enhances proliferation, invasion and EMT of tumor cells through the mTORC1 signaling pathway.

## INTRODUCTION

Nasopharyngeal carcinoma (NPC) is endemic in Southern Chinese and Southeast Asian population. Patients tend to present at the late or terminal stage at diagnosis due to its insidious onset and nonspecific early symptoms [1-3]. NPC has the highest metastasis features among head and neck cancers, with approximately 75–85% of patients have regional lymph node metastasis, and 15-19% of patients develop distant metastasis [4, 5]. Although NPC is sensitive to radiotherapy (RT) and the addition of chemotherapy to RT works effectively, treatment failure for NPC remains quite frequent, with rate of approximately 20% for distant metastasis [6, 7]. Over the past decades, the prognosis for NPC is still poor with a 5-year survival rate range from 50% to 70% for patients in advanced stage [8]. Therefore, identifying important biomarkers and understanding its molecular mechanisms to regulate the invasion and metastasis of NPC is urgently needed. Bone Morphogenetic Proteins (BMPs) are multi-functional secreted cytokines which belong to the transforming growth factor-beta (TGF-β) superfamily. To date, more than 20 BMPs have been identified in humans. Originally discovered by their ability to induce formation and development of bone and cartilage, these BMPs are now considered to play important roles in regulating cell differentiation, proliferation, motility, and survival [9-13]. It has been demonstrated that BMPs have an impact in cancer, but so far most of the studies have led to contradictory results implicating these molecules function as both suppressors and promoters of tumors in a context dependent manner, with a bi-directional characteristics in cancer that are similar to those of TGF-β [14-16].Therefore, the exact role of BMPs in cancer pathogenesis remains unclear. BMP2, one of isoforms of BMPs, had been shown to be overexpressed in several types of cancers including hepatocarcinoma [17, 18], non-small lung cancer [19-22], breast cancer [23-25], gastric [26-28], colon cancer [29, 30], bladder carcinoma [31], oral squamous carcinoma [32], prostate cancer[33], liposarcomas [34] and ovarian cancer [35]. The aberrant expression of BMP2 in above studies is correlated with the proliferation, differentiation, apoptosis, invasion and migration processes of cancer cells and thus may be regarded as an oncogene. However, some data revealed an opposite role of BMP2 in tumors. BMP2 was reported to function as potent tumor suppressors in breast cancer, gastric carcinoma, colorectal cancer, hepatocellular carcinoma and osteosarcoma, in which BMP2 suppress tumor growth by reducing the gene expression of tumorigenic factors and inducing the differentiation of cancer stem cells (CSCs) [36-39]. These results suggest that BMP2 may act as tumor suppressors or oncogene, depending on the cell type and tissue context.

To the best of our knowledge, no study has investigated the expression pattern and biological role of BMP2 in NPC. In our previous study, we characterized mRNA expression profiles of human NPC cell lines C666-1, CNE2 and non-neoplastic cell line NP69, immortalized from human nasopharyngeal epithelial cells, by RNA sequencing (RNA-seq) (unpublished data). Among the significantly differentially expressed genes, BMP2 was one of the dramatically upregulated genes observed in C666-1 and CNE2 cells compared with NP69 cells (logFC = 4.2 and 4.9, respectively, Supplementary Table 1). BMP2 mRNA overexpression was further validated in NPC biopsies by using quantitative real-time PCR (qRT-PCR).

In this research, we demonstrated that BMP2 was up-regulated in NPC compared with non-cancerous nasopharynx tissues and was closely related to advanced clinical stage, distant metastasis and poor survival of NPC patients. Additionally, we found that overexpression of BMP2 promoted proliferation, invasion and EMT of NPC cells. Furthermore, BMP2 could increase the phosphorylation of key factors in mTORC1 pathway including mTOR, S6K and 4EBP1. Our findings suggest that BMP2 promotes cancer aggressiveness through activation of mTORC1 pathway in NPC cells.

## RESULTS

### BMP2 is up-regulated in NPC tissues and cells

**Figure 1:**
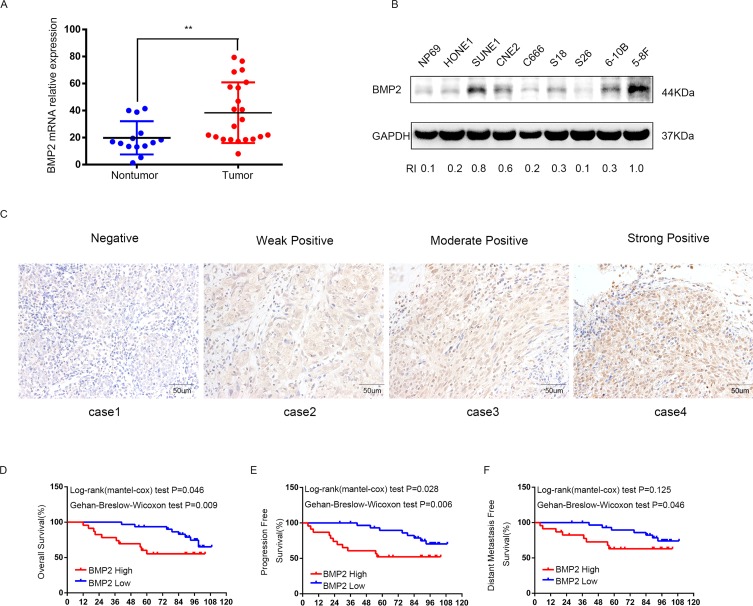
The expression of BMP2 is up-regulated in NPC and correlates with poor prognosis (**A**) qRT-PCR analysis showed that the expression of BMP2 mRNA was significantly higher in NPC tumor tissues (n=22) than non-cancerous nasopharyngitis tissues (n=14) (** p<0.01, independent Student's t-test). (**B**) The expression level of BMP2 in NPC cell lines (HONE1, SUNE1, CNE2, C666-1, S18, S26, 6-10B and 5-8F) and the normal epithelial NP69 was analyzed by Western blot. NP69 showed a relative low level of endogenous BMP2 protein expression and the protein expression level of BMP2 was obviously higher in S-18 and 5-8F cells with high tumorigenic and metastatic potential compared with their paired subclones S-26 and 6-10B cells with low tumorigenic and metastatic potential. (**C**) BMP2 immunostaining of representative images of NPC patients with different IHC scores. (**D**, **E**, **F**). Patients with high expression of BMP2 had shorter overall survival (OS) (**D**), progression-free survival (PFS) (**E**), and distant-metastasis-free survival (DMFS) (F) than those with low levels of BMP2.

The mRNA level of BMP2 was evaluated in 22 NPC tissues and 14 non-cancerous nasopharyngitis (NP) tissues by qRT-PCR. The result showed that the expression of BMP2 was significantly up-regulated in tumor tissues compared with non-tumor tissues (P<0.01) (Fig. 1A). The protein expression level of BMP2 in immortalized nasopharyngeal epithelial cell line NP69 and NPC cell lines (HONE1, SUNE1, CNE2, C666-1, S18, S26, 6-10B and 5-8F) were measured by using western blot analysis. It was showed that NP69 has a relative low level of endogenous BMP2 protein expression compared with NPC cell lines (Fig. 1B). Interestingly, the protein expression level of BMP2 was obviously higher in S-18 and 5-8F cells with high tumorigenic and metastatic potential compared with their paired subclones S-26 and 6-10B cell lines with low tumorigenic and metastatic potential (Fig. 1B).

### BMP2 protein overexpression correlates with disease progression and distant metastasis of NPC patients

To investigate the clinical significance of BMP2 expression in NPC patients, 54 formalin-fixed paraffin-embedded NPC samples were detected by immunohistochemical (IHC). The IHC staining scores for BMP2 in these NPC samples range from 0 to 3 and tissue representative images of those NPC patients with different IHC scores are showed in Fig. 1C. NPC samples were defined as high and low BMP2 expression using the median IHC score as the cutoff point. 23 out of the 54 (42.6%) NPC samples were identified as high BMP2 expressing (staining index > 1). The association between the expression of BMP2 and patients' clinical characteristics was analyzed by Chi-square test, which was summarized in Table 1. As shown, high expression of BMP2 was significantly correlated with larger tumor size (P = 0.038), lymph node metastasis (P = 0.008), advanced clinical stages (P = 0.000), distant metastasis rate at diagnosis (P=0.01) and bone metastasis (P=0.028). No significant association was found between BMP2 expression and other clinic-pathological features. To explore the prognostic value of BMP2 protein in NPC patients, Kaplan–Meier method and the log-rank test survival analysis were used. Results showed that patients with a higher expression level of BMP2 had a significantly poorer overall survival (OS), progression-free survival (PFS), and distant-metastasis-free survival (DMFS) than those with low level of BMP2 (P<0.05; Fig. 1D, E and F). These results suggested that BMP2 was involved in NPC prog-ression.

**Table 1:** Association of BMP2 expression and patient clinicopathological characteristics in NPC tissues

Characteristics	No	BMP2 expression level		P value (Chi-square test)
Gender		Low	High	
Male	34	19(61.3%)	15(65.2%)	0.768
Female	20	12(38.7%)	8(34.8%)	
Age				
< 44	24	14(45.2%)	10(43.5%)	0.902
≥ 44	30	17(54.8%)	13(56.5%)	
DNA copy				
≤ 4000	34	21(67.6%)	13(56.5%)	0.399
> 4000	20	10(32.4%)	10(43.5%)	
EA/IgA				
< 1:10	11	5(16.1%)	6(26.1%)	0.369
≥ 1:10	43	26(83.9%)	17(73.9%)	
VCA/IgA				
< 1:80	8	3(9.7%)	3(9.7%)	0.369
≥ 1:80	46	28(90.3%)	28(90.3%)	
T stage				
T1	6	4(12.9%)	2(8.7%)	0.038*
T2	11	7(22.6%)	4(17.4%)	
T3	24	17(54.8%)	7(30.4%)	
T4	13	3(9.7%)	10(43.5%)	
N stage				
N0	8	6(19.4%)	2(8.7%)	0.008*
N1	23	12(38.7%)	11(47.8%)	
N2	17	13(41.9%)	4(17.4%)	
N3	6	0(0)	6(26.1%)	
Clinical staging				
I	2	1(3.2%)	1(4.3%)	0.000*
II	4	2(6.5%)	2(8.7%)	
III	33	26(83.9%)	7(30.4%)	
IV	15	2(6.4%)	13(56.6%)	
Distant metastasis (at diagnosis)				
Yes	12	3 (9.7%)	9 (39.1%)	0.010*
No	42	28 (90.3%)	14 (60.9%)	
Bone metastasis				
Yes	9	2(6.5%)	2(6.5%)	0.028*
No	45	29(93.5%)	29(93.5%)	

### BMP2 enhances NPC cell proliferation

**Figure 2:**
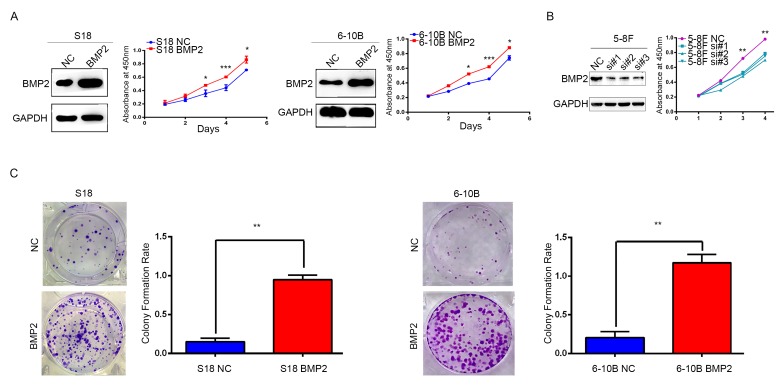
BMP2 enhances NPC cells growth (**A**) The over-expression efficiency of BMP2 in S18 and 6-10B cells were confirmed by Western blot, respectively. And CCK-8 assays showed that BMP2 could significantly promote proliferation of S18-BMP2 and 6-10-BMP2 cells compared with the controls. (**B**) BMP2 knockdown in 5-8F cell via three specific siRNA was confirmed by Western blot and CCK-8 assays showed that BMP2 knockdown reduced the proliferation of 5-8F cell. (**C**) In colony formation assays, the number of colonies was significantly higher in the S18-BMP2 and 6-10B-BMP2 cells than the controls.

S18 and 6-10B cells were transfected with recombinant lentivirus expressing BMP2 or a negative control lentivirus (NC), and BMP2 expression was confirmed by western blot analysis (Fig. 2A). In CCK-8 assays, BMP2 overexpression significantly accelerates the proliferation of S18-BMP2 and 6-10-BMP2 cells compared with the controls (Fig. 2A). To further investigate the function of endogenous BMP2 in NPC cells, BMP2 was silenced in 5-8F cell by each of three specific siRNA against BMP2 as well as with one control (NC) siRNA. CCK-8 assays showed that BMP2 knockdown significantly reduced cell proliferation of 5-8F cell (Fig. 2B). Moreover, colony numbers were much increased in BMP2 overexpressed cells than those control cells in colony formation assays (Fig. 2C). These results indicated that BMP2 could enhance NPC cell proliferation.

### Upregulation of BMP2 promoted NPC migration and invasion *in vitro*

**Figure 3:**
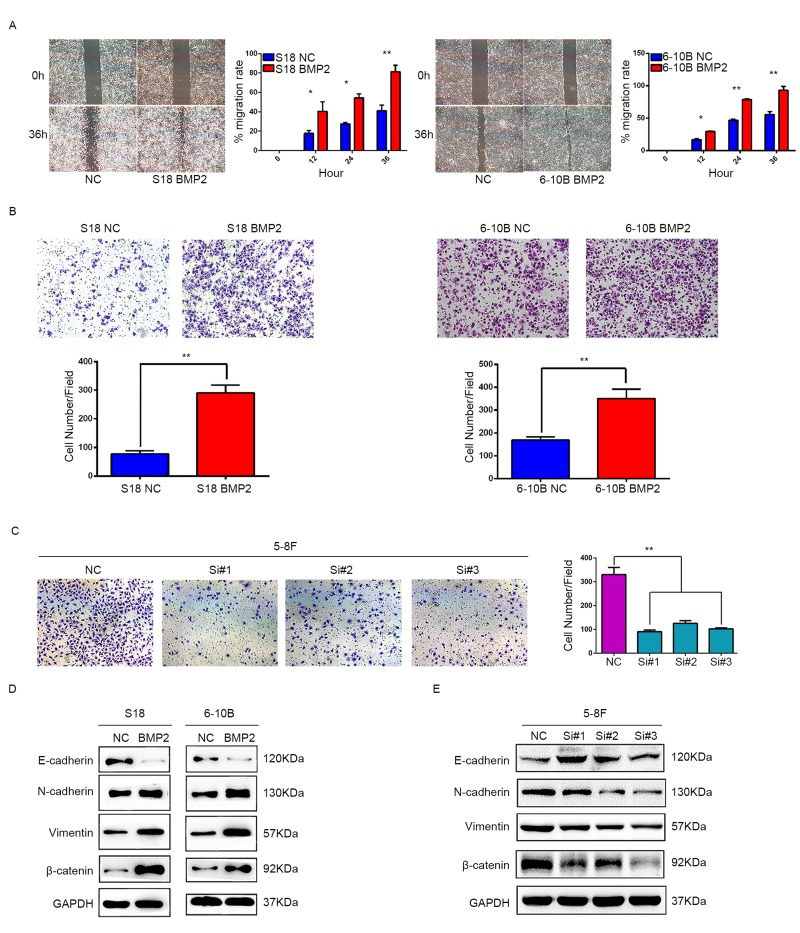
BMP2 promotes NPC cell migration, invasion and epithelial-mesenchymal transition (**A**) Wound-healing assays demonstrated that BMP2 upregulated cells had higher migration rate than control cells. (**B**) Matrigel invasion chambers were used to evaluate the ability of NPC cell invasion. Upregulation of BMP2 significantly increased the number of invaded cell in S18 and 6-10B compared with the controls. (**C**) silencing expression of BMP2 in 5-8F much reduced the invaded cell numbers. (**D**) Western blotting revealed that overexpression of BMP2 in S18 and 6-10B cells decreased the expression of epithelial makers (E-cadherin) and increased the expression of mesenchymal markers (N-cadherin, Vimentin and β-catenin) in comparison with the controls. (**E**) Western blotting showed that knockdown of BMP2 increased the expression of E-cadherin and decreased the expression of N-cadherin, Vimentin and β-catenin.

Since a relationship between BMP2 overexpression and NPC metastasis was supported by clinical evidence, we then investigated the role of BMP2 in migration and invasion of NPC cells. In wound healing assay, cell migration rate was markedly increased in the S18-BMP2 and 6-10B-BMP2 cells compared with control cells (Fig. 3A). In matrigel coated transwell assay, upregulation of BMP2 significantly increased the number of invaded cell in S18 and 6-10B cells by approximately triple and twice, respectively (both P<0.01) (Fig. 3B) and knockdown of BMP2 in 5-8F cell decreased the invaded cell numbers from 330 to 91, 125 and 102, respectively (Fig. 3C). These results showed that upregulation of BMP2 promoted NPC migration and invasion in vitro.

### Upregulation of BMP2 induces NPC cell epithelial-mesenchymal transition (EMT)

EMT is the first and most critical step in tumor metastasis. To address the significance of BMP2 in NPC cell metastasis in vitro, we next sought to identify whether BMP2 is involved in the EMT process in NPC cells. Western blot assays showed that BMP2 overexpression was able to trigger EMT featured by the gain of mesenchymal markers, such as N-cadherin, vimentin and β-catenin, and the loss of epithelial marker E-cadherin in S18 and 6-10B cells (Fig. 3D). The opposite results were obtained in 5-8F cell following BMP2 knockdown (Fig. 3E). These results demonstrated that upregulation of BMP2 induced EMT of NPC cells.

### BMP2 induces proliferation and invasion of NPC cells through the mTORC1 signaling pathway

**Figure 4:**
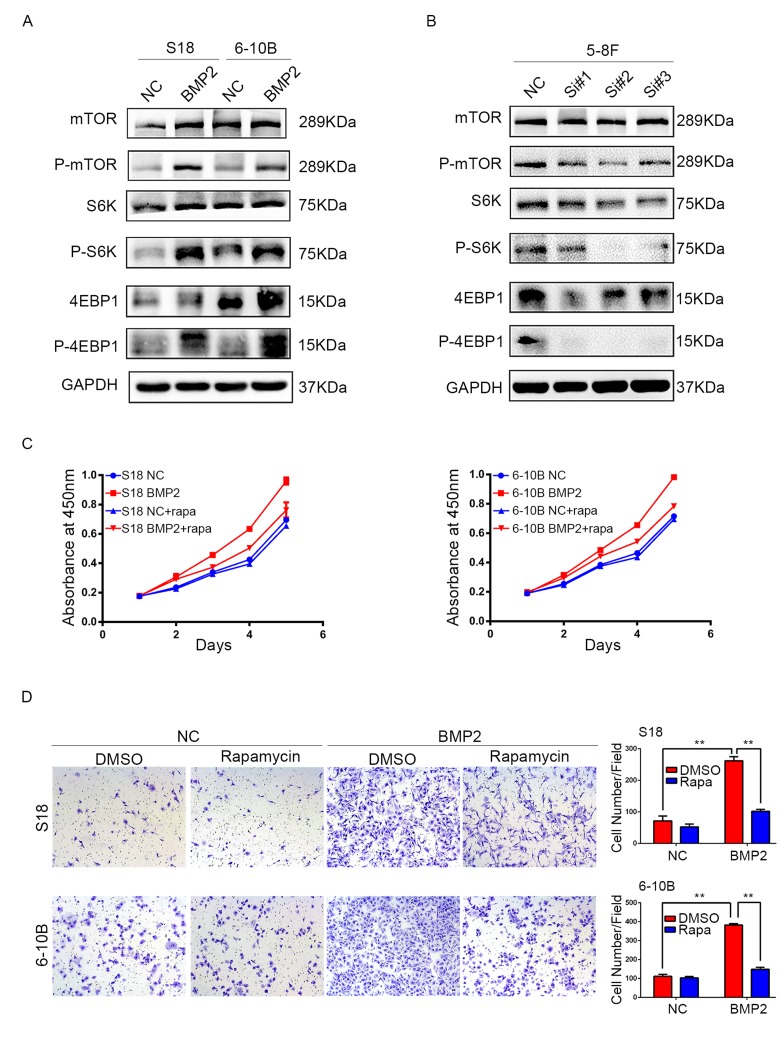
BMP2 activates mTOR signal pathway in NPC cells (**A**) Western blotting analysis revealed that in S18-BMP2 and 6-10B-BMP2 cells, the expression of phosphorylated (active) mTOR, P70S6K and 4EBP1 (p-mTOR, p-p70S6k and p-4EBP1) were upregulated compared to those control cells. (**B**) The expression of p-mTOR, p-p70S6k and p-4EBP1 were downregulated in 5-8F knockdown BMP2 cell. (**C**) Proliferation abilities of S-18 and 6-10B cells expressing BMP2 or the empty vector was evaluated by CCK8 assay after pretreatment with or without 20 nM of rapamycin. (**D**) Invasive abilities of S-18 and 6-10B cells expressing BMP2 or the empty vector was evaluated by transwell assay after pretreatment with or without 20 nM of rapamycin.

Since mTOR pathway regulates proliferation, metastasis and is frequently activated in many kinds of cancers including NPC, we examined the correlation between BMP2 expression and the key substrates of mTOR signaling pathway using western blotting assay. In S18-BMP2 and 6-10B-BMP2 cells, the expression of phosphorylated (active) mTOR, P70S6K and 4EBP1 were upregulated compared with those control cells, but no obvious changes were seen in the total amounts of mTOR, P70S6K and 4EBP1 (Fig. 4A). Conversely, the levels of phosphorylated mTOR, P70S6K and 4EBP1 were downregulated in BMP2 knockdown 5-8F cell (Fig. 4B). The results indicate that BMP2 activate mTORC1 pathway in NPC cells.

To further determine the importance of mTORC1 signaling pathway in NPC proliferation and invasion, S18-BMP2 and 6-10B-BMP2 cells were treated with mTORC1 pharmacological inhibitor (rapamycin). Results showed that treatment of rapamycin (20nM) suppressed the ability of BMP2 to enhance cell growth and invasion (Fig. 4C and D).

These findings suggest that the BMP2 promotion of proliferation and invasion of NPC cells depends on the mTORC1 signaling pathway.

## DISCUSSION

In our previous study with aim to characterize aberrant transcript expression that contribute to the NPC oncogenesis, RNA-seq was performed to discriminate gene expression profiles in NPC cell lines CNE2 and C666-1 and an immortalized nasopharyngeal epithelial cell line NP69 using an Illumina Hiseq 2500 platform (unpublished). 1323 and 1106 differentially expressed mRNAs (p < 0.05) were identified in C666-1 and CNE2 cells when compared with NP69 cells, respectively. One of the most highly up-regulated genes was the BMP2 gene, which showed dramatically elevated expression in C666-1 and CNE2 compared with NP69 (logFC = 4.2 and 4.9, respectively, Supplementary Table 1). Subsequently, overexpression of BMP2 mRNA in NPC biopsies was confirmed by quantitative RT-PCR analysis in snap-frozen human NPC and non-cancerous nasopharynx tissues. The protein expression levels of BMP2 in NPC cells and biopsies were evaluated by using western blot and Immunohistochemistry staining methods. The protein expression level of BMP2 was lowest in immortalized nasopharyngeal epithelial cell line NP69 compared with NPC cell lines, and obviously higher in S-18 and 5-8F cells with high metastatic potential compared with their paired subclones S-26 and 6-10B cells with low metastatic potential. According to the correlation analysis between BMP2 expression level and clinical-pathological features of NPCs, BMP2 overexpression was significantly positively correlated with tumor size, lymph node metastases, distant metastasis and clinical stage in NPC patients. Notably, the bone metastasis rate was as high as 30.4% (7/23) in patients with high BMP2 expression, but only 6.5% (2/31) in BMP2 low-expression groups. Furthermore, the association of BMP2 overexpression with worse clinical outcome, in terms of OS, PFS and DMFS, was confirmed by using Kaplan–Meier and log-rank test survival analysis.

In terms of its cellular effects, BMP2 overexpression promotes cell proliferation, colony formation, migration and invasion in NPC cells. Upregulation of BMP2 increased N-cadherin, vimentin and β-catenin expression and decreased E-cadherin level in S-18 and 6-10B cells. Reversely, knockdown of endogenous BMP2 reduced cell invasive capability and inhibit EMT phenotype in highly aggressive 5-8F NPC cells. Both the in vitro experiments and the clinical evidence support that BMP2 over-expression promotes tumor proliferation and aggressiveness of NPC.

mTOR signaling pathway plays a central role in regulating cell proliferation and metastasis, and drives EMT in tumor cells [40]. Mammalian target of rapamycin (mTOR), an evolutionarily conserved and ubiquitous serine/threonine kinase, plays a central role in regulating cell proliferation, apoptosis, autophagy and migration, being considered a hotspot target for cancer therapy [41]. mTOR can form two distinct multiprotein complexes, mTOR complex 1 and 2 (mTORC1 and mTORC2). mTORC1 activation results in phosphorylation of 4EBP1 and S6K1, while mTORC2 promotes Akt phosphorylation [42]. mTORC1 is activated by growth factors and nutrients, via PI3K, MAPK or AMPK, and as such is able to regulate cell growth, proliferation and survival [43, 44]. Mounting evidence shows that mTORC1 is crucial in the invasion and migration of multiple cancer cells [45]. It was reported that mTOR signaling pathway is frequently activated and correlated with a poor prognosis in NPC [46-48]. To explore the underlying molecular mechanisms of the aggressiveness promoting role of BMP2 in NPC cells, the effect of BMP2 expression on mTOR signal pathway was measured. In the present study, it was determined that the expression of phosphorylated (activated) proteins in the mTOR pathway, including p-mTOR, p-70S6, and p-4EBP1 were increased in BMP2 over-expression NPC cells with no obvious changes in the total amounts of these proteins. Conversely, the opposite results were obtained when endogenous BMP2 expression was knockdown by siRNAs in 5-8F NPC cells. Moreover, mTORC1 inhibitor rapamycin treatment abolished the effects of BMP2 on cell growth and invasion ability in BMP2-over-expressing S18 and 6-10B cells. Collectively, these findings support that BMP2 promote tumor proliferation and invasion via activation of mTORC1 signalling pathway in NPC cells.

BMPs have a multiplicity of effects that range from controlling important steps of embryonic development to regulating growth, differentiation and apoptosis of different cell types. BMPs mediate their biological effects by binding to type I/II receptor to form a heterotetrameric complex that contain serine/threonine kinase domains. Upon BMP binding, the receptor complex mediates intracellular signaling via phosphory-lating Smad. Then, these phosphorylated Smads associate with Smad4 and translocate to the nucleus to drive the target genes expression [49, 50]. In addition to the canonical SMAD pathway, BMPs activate several intracellular pathways include PI3-kinase, Cdc42, Erk, TAK-p38, MAPK, Wnt, which form a complex network of molecular signals regulating multiple cellular responses [51, 52]. Although dysregulation of BMPs signaling has been linked to various types of cancers, the relationship between abnormal activation of these signaling pathways and tumorigenesis is not clear, due to conflicting findings showing BMPs promote or inhibit tumorigenesis in a cell-type and context-dependent manner.

During development, BMP2 induces the PI3K/ mTOR signaling pathway to regulate stem cell differentiation [53]. It was reported that BMP2 induces the phosphorylation of mTOR and p70S6 kinase in lung cancer cell lines through a Smad 1/5–independent mechanism [54] and promotes motility and invasion of chondrosarcoma cells, gastric cancer cells and pancreatic cancer cells by activating PI3K/Akt signaling pathway [55-57]. Our findings from this study are generally in consistent with the previous studies. However, another study reported that BMP2 exerts an inhibitory effect on the growth and migration of hepato-cellular carcinoma cells via a blockade of PI3K/AKT signaling [39]. These reports indicate that the expression pattern and biological function of BMP2 in cancer might be determined by context-dependent factors.

A close association of BMP2 expression with bone metastasis of NPC has be verified in the present study, which indicates that BMP2 expression in primary tumor seems to correlate with the risk for accelerated bone metastasis formation in NPC patients. NPC has a special predilection to form bone metastases. Previous studies demonstrated that the bones account for 50% cases of metastasis in NPC, and metastasis to bone is associated with a poor prognosis and survival outcomes [58].Therefor, identifying the predictors of bone metastasis and giving early intervention are important research direction. BMP2, BMP4 and BMP7 are involved in osteoblast lineage differentiation [59, 60] and have the ability to induce new bone formation [61]. Therefore, BMPs expression in the primary tumor might have be an important factor in the progression of bone metastases of cancer. Expression of BMP2 was significantly higher in bladder urothelial carcinoma cases with bone metastasis [62]. Increased expression of BMP7 was also identified in bone metastatic breast cancer and prostate cancer [63, 64]. Our findings indicate that BMP2 might be a potential biomarker in predicting bone metastasis of NPC.

In conclusion, our studies demonstrated for the first time that BMP2 is upregulated in NPC and induces proliferation, migration, invasiveness and EMT of NPC cells. Furthermore, we revealed that BMP2 promotes proliferation and invasion of NPC cells via mTORC1-S6K signaling pathway. We propose that BMP2 may serve as a prognostic biomarker and therapeutic strategy in the management of NPC.

## MATERIALS AND METHODS

### Patients and tissue samples

Twenty-two fresh NPC tissues and fourteen non-cancerous nasopharyngitis (NP) tissues were collected from the department of Nasopharyngeal Carcinoma, Sun Yat-Sen University Cancer Center (SYSUCC). Each of those tissues was soaked in RNA-later in 4°C overnight, and then moved to −80°C to store until use for qRT-PCR assay. Another fifty-four archival formalin-fixed, paraffin-embedded primary NPC tissues which were used for the immunohistochemistry assay were obtained from the Department of Pathology in the SYSUCC. All of NPC samples had been confirmed pathologically between 2007 and 2008 and none of those patients received treatment before definitive diagnosis, including radiotherapy and chemotherapy. Of the fifty-four NPC patients, the median age was forty-four which ranged from 17 to 70, including thirty-four males (63%) and twenty females (37%). Clinical and pathological information of those NPC patients were listed in Table 1. Our study was approved by Research Ethics Committee of SYSUCC and the informed consent had written by all patients.

### Cell lines

In this study, eight human NPC cell lines containing HONE1, SUNE1, CNE2, C666-1, S18, S26, 6-10B and 5-8F as well as one nasopharyngeal normal epithelium cell line NP69 were used for detecting the expression of BMP2. Those NPC cells were maintained in Roswell Park Memorial Institute (RPMI) 1640 medium (Gibco, Shanghai, China), which were added additional 10% fetal bovine serum (Gibco, US). The NP69 cell was grown in defined-KSFM medium which was rich in EGF (Invitrogen, Carlsbad, CA). All above cell lines were cultured at 37°C and 5% CO_2_.

### Antibodies

Rabbit anti-BMP2 antibody was obtained from ABclonal Technology. Mouse anti-GAPDH (for normalized control), rabbit anti-mTOR, rabbit anti-P-mTOR, rabbit anti-S6K, rabbit anti-P-S6K (T398), rabbit anti-4E-BP1 and rabbit anti-P-4E-BP1 were from Cell Signaling Technology (CST). Mouse anti-E-cadherin, anti-N-cadherin, anti-β-catenin and anti-vimentin were from BD Biosciences. Both anti-rabbit and anti-mouse labeled with horseradish peroxidase (HRP) were from CST.

### Quantitative real-time PCR (qRT-PCR)

Each tissue was fragmented with Trizol (Invitrogen, USA) to extract RNA following the manufacturer's instructions. Total RNA (1μg) was used as template for synthesis cDNA under the instruction of PrimeScript RT reagent Kit (Promega, Madison, WI, USA). Then qRT-PCR was performed with 15ul total volume, including 1ul of cDNA, 0.5ul of each primer and 13ul of GoTaq qPCR Master Mix (Invitrogen, USA). In the end, the expression of BMP2 of each sample was carried out using Bio-Rad CFX96™ Real-Time PCR Detection System with GAPDH as a normalizing control. The specific primer sequences of BMP2 and GAPDH as follow:

BMP2 sense 5′- ACCCGCTGTCTTCTAGCGT-3′;

BMP2 anti-sense 5′- TTTCAGGCCGAACATGCTGAG-3′;

GAPDH sense 5′- CTCCTCCTGTTCGACAGTCAGC-3′;

GAPDH anti-sense 5′- CCCAATACGACCAAATCCGTT-3′.

The relative expression of BMP2 was given by 2^−ΔΔCt^ equation.

### Immunohistochemistry (IHC) staining

Those formalin-fixed, paraffin-embedded primary NPC tissues were sectioned (4 μm) and baked at 60°C overnight. Then IHC study was performed as described previously [65]. Two pathologists evaluated and scored the staining results of BMP2 independently and the intensities of immunostaining were defined as: 0 (negative), 1 (weakly positive), 2 (moderately positive) and 3 (strongly positive).

### Western blotting

As described previously [66], all of cells were lysed by RIPA and the protein of each cell was collected, measured and denatured. Then 10% SDS poly-acrylamide gel electrophoresis and PVDF membrane (GE Healthcare Life Sciences, UK) were used sequentially to separate and transfer those protein. TBST containing 8% nonfat milk blocked the mem-branes at room temperature (RT) for 2 hours. And then the membranes were incubated with primary antibodies at 4°C for 16 h, incubated secondary antibodies labeled with horseradish peroxidase (HRP) for 1h at RT and detected by electrochemiluminesce (ECL) system (Tanon, China).

### Lentivirus transduction

For BMP2 overexpression study, S18 and 6-10B cells stably expressing BMP2 were obtained by infecting with BMP2 expression lentiviral vector (GenePharma, Shanghai, China) and selecting with 2 μg/mL puromycin for 2 weeks according to the manufacturer's instructions. So as the control cells infecting with empty vector (GenePharma, Shanghai, China).

### siRNA interference

The siRNA1321, siRNA1498, siRNA1628 and the negative control (NC) siRNAs of BMP2 were designed and synthesized by GenePharma (Shanghai, China). When 5-8F cell in the six-well plate reached 50%, 100nM siRNA1321, siRNA1498, siRNA1628 and NC of BMP2 were transfected with Lipofectamine™ 3000 (Invitrogen, USA), respectively, according to the manufacturer's instructions. Those transfected cells were incubated for 48 h and then were measured by western blotting.

### CCK8 assay

Cell viability was detected by CCK8 Cell Counting Kit (JingXin Biological Technology, Guangzhou, China). Cells were plated and cultured in 96-well plates with 1.0×10^3^ cells/well. The detection was done at a regular time every day. Briefly, the tested wells were added 10 μl CCK8 respectively and incubated at 37°C for 2 hours. Then the OD value was measured at 450 nm by a microplate reader (SpectraMax® M5 Multi-Mode Microplate Reader; Molecular Devices LLC, Sunnyvale, CA, USA). To test the effect of mTOR activation on the cell proliferation induced by BMP2 expression, NPC cells were pretreated with Rapamycin (20 ng/ml) (Selleck Chemicals, USA) for 30 min before performing the CCK8 assays.

### Colony formation assay

S18-BMP2, 6-10B-BMP2 and their control cells were seeded in six-well plates with 500 cells/well respective-ly. Two weeks later, the colonies were fixed with methanol for 10 min, stained with 0.5% crystal violet for 15 min and then counted under a dissection microscope.

### Migration and invasion assays

Migration and invasion assays were explored as described previously [65]. The scratch wound-healing assay was performed to detect migration ability of BMP2-overexpression cells. Briefly, cells were plated and cultured in 6-well plate until reached sub-confluence, then the medium without FBS was used in every plate. A linear scratch wound was produced with a sterile plastic micropipette tip. Washed wells by sterile water before the wound was observed and photographed every 12 hours.

Matrigel-coated chamber (BD Biosciences) was used to detect invasion ability of cells. NPC cells were trypsinized, resuspended by serum-free medium and counted to make sure the concentration of each cell was 2.5×10^5^ cells/ml. Chambers were inserted into a 24-well plate with 20% FBS RPMI-1640 medium (700μl/well). Then 200ul of above cells (2.5×10^5^ cells/ml) were seeded in the upper chamber and the plate was incubated for 20 hours. Those non-invading cells of upper chamber were removed by a cotton swab thoroughly. Invasive cells in the filters were immersed in 100% methanol for 10 min and then stained with 0.1% crystal violet (Weijia Biology Science and Technology Co, Ltd) for 20 min at RT. Five random fields of per chamber were counted under a microscope. To test the effect of mTOR dephosphorylation on the cell invasion induced by BMP2 expression, NPC cells were pretreated with Rapamycin (20 ng/ml) (Selleck Chemicals, USA) for 30 min before performing the invasion assays.

Experiments were repeated three times.

### Statistical analysis

Statistical analysis was performed with SPSS version 17.0 software (SPSS, Inc., Chicago, IL, USA). Chi-square test was applied to analysis the correlation between BMP2 expression and clinical characteristics of patients with NPC. In this study, we estimated overall survival (OS), progression-free survival (PFS), and distant-metastasis-free survival (DMFS) by the Kaplan Meier method and the log-rank test. Student's t test was performed to compare data of two independent groups. P<0.05 was considered statistically significant (*P < 0.05; **P < 0.01; ***P < 0.001).

## SUPPLEMENTARY MATERIAL TABLE


