# Supplementary material for: BMP2 promotes proliferation and invasion of nasopharyngeal carcinoma cells via mTORC1 pathway
Source: Aging (Albany NY). 2017 Apr 28;9(4):1326–39. doi: 10.18632/aging.101230 (PMC5425130; doi:10.18632/aging.101230)
Supplement: Supplementary file 1 [file aging-09-1326-s001.pdf]

SUPPLEMENTARY MATERIAL

Supplementary Table 1. BMP2 was up-regulated in C666-1 and CNE2 cells compared with NP69 cells in RNA-Seq data.

Gene	Mean expression value			$\log_2$(fold change)	*P*-value
	NP69	C666-1	CNE2		
BMP2	0.7020		21.2442	4.9194	0.0003
	0.8166	14.5914		4.1593	0.0077
